# We can make it happen

**DOI:** 10.4102/phcfm.v9i1.1645

**Published:** 2017-11-22

**Authors:** Per Kallestrup

**Affiliations:** 1 Skødstrup Lægepraksis, Skødstrup, Denmark; 2 Department of Public Health, Aarhus University, Denmark

A review of Jan De Maeseneer’s book: *Family Medicine and Primary Care – At the Crossroads of Societal Change*.

By Per Kallestrup, family physician in Skødstrup Lægepraksis and associate professor in Global Health, Aarhus University, Denmark.

All primary care professionals do at times feel overburdened, losing faith in their own capabilities and deprived of hope in their health systems’ ability to provide care to meet the needs of their populations. The ‘inverse care law’ is universal and an inevitable part of the agenda, when it comes to the everyday challenges of primary care providers.

Nevertheless, resilience and ‘doing the right thing, particularly on a hard day’ are exactly among the inspirational characteristics as well as unsung motivating rewards of first-line health care professionals. Moreover, when this incredible individual strength and perseverance is organised into community health teams, sustainable delivery of ‘health for all’ becomes feasible.

Jan De Maeseneer’s book, *Family Medicine and Primary Care – At the Crossroads of Societal Change*, is an inspirational personal tale of the remarkable developments of Belgian, European, African and global family medicine and primary care over the last four decades since the Alma Ata Declaration. These developments are in many ways parallel to and coinciding with the remarkable roadmap of Jan’s career as a medical student, family physician, educator, academic, politician and visionary activist and cosmopolitan.

We are guided through the milestones in the history of family medicine and primary care as well as introduced to the main theories, paradigms and dilemmas shaping the landscape. Backgrounds are explored and explained and advice and direction given for as varied topics as social determinants of health and diversity, goal-oriented care and multimorbidity, diagnostic reasoning and dealing with uncertainty, availability of medicine and the pharmaceutical industry, developing quality of care, training of health professionals, in particular family physicians to fit the needs of populations and the organisation, as well as financing mechanism for primary care to deliver societal change for the better.

All chapters begin with an easily recognisable ‘patient story’. Real-life scenarios that form the introduction as well as backdrop to the societal and structural elements discussed. This way the reader is encouraged to reflect on and follow the holistic principles of person-centred and population-centred primary care integrating all elements in a continuum and adapted to the local context.

It is through these testimonies that the real value of this book is revealed: the importance of trusted relationships to individuals and communities, dedicated and effective professionals, who are able to think and act ‘glocal’ as tireless, responsible ambassadors seeking the ‘quadruple aim’ and accessible, inclusive primary care of good quality as the hallmark for social cohesion, stability and peace.

Jan De Maeseneer’s restless and generous ability to not only master words and theories but demonstrate in (inter)actions and constructs how to ‘practice, teach, change and live’ family medicine and primary care is truly enlightening and inspiring.

With authenticity and integrity through the decades, he has been a practising clinician and has participated in the innovative experiments of a dynamic community health centre ‘Botermarkt’ in Ledeberg, a deprived area of the city of Ghent. Through the book, it is clear that this ‘living laboratory’ has provided him the fuel to engage in the ‘bigger picture’.

From this local base and in parallel to his dedication there, he has taken an extraordinarily active part and demonstrated leadership in a multitude of ways for the educational, scientific and organisational development of family medicine and primary care regionally, nationally and globally. Yet, the descriptions of these activities – like the patient vignettes – serve as examples of how it is important and simultaneously rewarding as an individual practitioner to maintain a broader focus with constant vigilance and commitment.

Read Jan De Maeseneer’s book as the right thing to do on a hard day (as well as any other day). If you sometimes feel ‘down and out’ when facing the often awkward and messy (yet, wonderful) realities of front line primary care, this book is so full of optimism, enthusiasm and energy – hope to carry on. We can make it happen!

